# Intraosseous Cavernous Hemangioma in the Mandible: A Case Report

**DOI:** 10.4317/jced.52864

**Published:** 2017-01-01

**Authors:** Bilgir Elif, Yildirim Derya, Kocer Gulperi, Bozova Sevgi

**Affiliations:** 1 PhD, Research Assistant; Suleyman Demirel University, Faculty of Dentistry, Department of Dentomaxillofacial Radiology, Isparta, Turkey; 2 DDS, PhD, Associated Professor; Suleyman Demirel University, Faculty of Dentistry, Department of Dentomaxillofacial Radiology, Isparta, Turkey; 3 DDS, PhD, Associated Professor; Suleyman Demirel University, Faculty of Dentistry, Department of Oral and Maxillofacial Surgery, Isparta, Turkey; 4 MD, Specialist Doctor of Medicine; Ministry of Health Turkish Public Hospitals Institution Association of Antalya Public Hospitals Serik State Hospital, Department of Pathology, Antalya/Turkey

## Abstract

Intraosseous vascular lesions are rare conditions. They are most commonly seen in the vertebral column and skull; nevertheless, the mandible is a quite rare location. In this report, we present a case of intraosseous cavernous hemangioma in the mandible and discuss the clinical and radiological features. A 28-year-old male patient attended to our clinic with a complaint of painless swelling of mandible. Clinical evaluation revealed a bone-hard, smooth-surfaced, immobile mass in the left mandibular lingual area. The patient was evaluated with panoramic and occlusal radiography and computed tomography. The lesion surgically excised and pathological examination revealed an intraosseous cavernous hemangioma. Follow-up imaging 1 year later with cone beam computed tomography revealed recurrence of the lesion. The conclusion of this paper; when a bone hard, well-shaped mass was seen in the mandible, the possibility of intraosseous hemangioma must be remembered and before surgical procedure detailed radiographic evaluation should be performed.

** Key words:**Hemangioma, intraosseous, mandible, cavernous, cbct.

## Introduction

Primary intraosseous hemangioma is a benign, slow growing neoplasm that comprises 1% of all benign tumors arising from the bones. Hemangiomas mostly arise from the soft tissues, and intraosseous hemangiomas are uncommon (1-4). The sites most commonly involved are the vertebral column and the calvarium in the maxillofacial region. They have been reported in the man-dible, maxilla, and the nasal bones (5-7). Intraosseous hemangioma usually occurs in adults, and many patients are in fifth decade of life, with a female predominance. The diagnosis can be difficult, and usually neither the history nor the physical findings lead the clinician to suspect an osseous cavernous hemangioma (1,5,8). They usually become symptomatic with slow-growing, painful swelling (3,8). The radiographic presentation of the cavernous hemangioma is nonspecific. The lesion appears as a radiolucency that could have unilocular or multilocular, reticulated, honeycomb, or sunburst appearance when viewed tangentially. These findings are generally called ‘sunburst appearance’ for skull hemangioma, ‘corduroy- like appearance’ for vertebral hemangioma, and ‘honeycomb appearance’ for rib hemangioma. Most cases of the bone hemangioma can be diagnosed with characteristic radiographic findings that reflect the formation of reactive spicula produced by the lesion. However, this classic feature may be absent in many cases, which present only as lytic or expanding dense bone masses (1,6,9). In this report, we presented a male patient on the third decade of life with intraosseous cavernous hemangioma in the mandibleand discussed the clinical and radiological features with characteristic radiographic findings.

## Case Report

**Figure 1:**
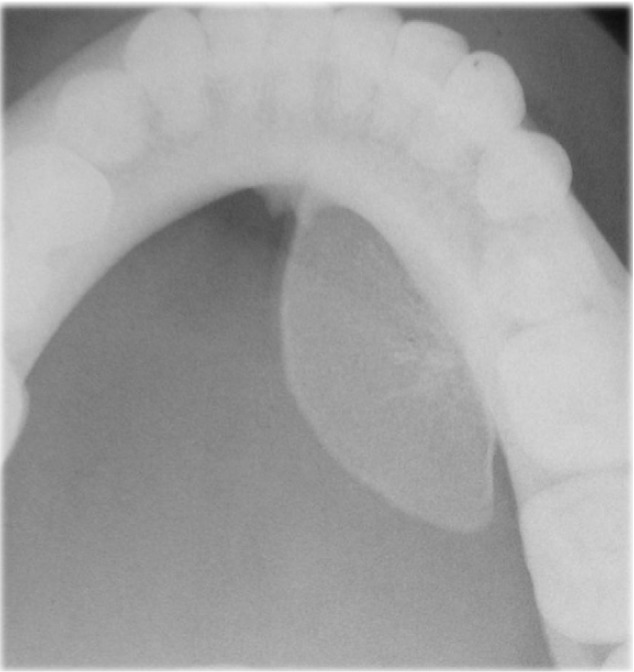
Occlusal radiograph revealed a well defined lesion surrounded by a sclerotic margin, containing reactive bone spicules which are characteristic for intraosseous cavernous hemangioma.

**Figure 2:**
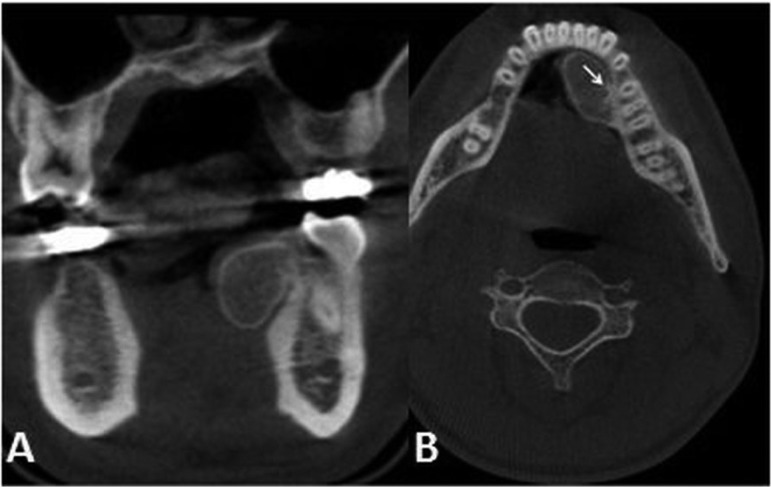
Cone beam computed tomography scan shows the intraosseous cavernous hemangioma. No signs of destruction of the adjacent tissue are present. A) Coronal section showing the bone mass originated from left lingual mandibular region. B) Axial section; the formation of reactive spicula produced by the lesion was indicated by the arrow.

**Figure 3:**
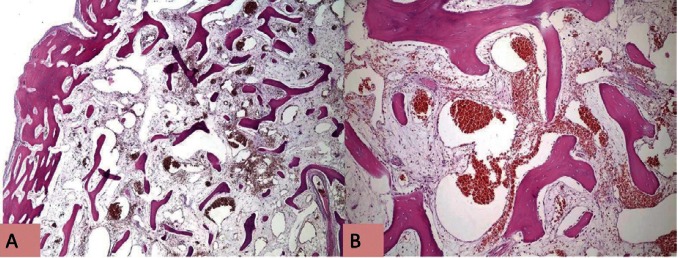
A,B) Histopathologic slide show that blood-filled vascular space between the bone trabeculae.

A 28-year-old male patient attended to our clinic with a complaint of painless swelling of mandible. The patient first noticed the swelling about two years ago when brushing his teeth. This swelling which has more than doubled in size since the difference has led to difficulty in speaking since 3 months. Intraoral examination revealed bone-hard, smooth-surfaced, immobile mass in the left mandibular lingual crest. Computed tomography (CT) that had taken at her previous visit at another hospital examined,maxillofacial computed tomography demonstrated a 21x13 mm bone mass originated from lingual cortex of mandible. Panoramic and occlusal radiography imaging performed. Panoramic radiograph of the patient did not reveal any pathology. Occlusal radiography revealed a well defined lesion with radiopaque contours, containing reactive bone spicules (Fig. 1). Preliminary diagnosis was made as torus or intraosseous cavernous hemangioma. The lesion surgically was excised and pathological examination revealed an intraosseous cavernous hemangioma. Follow-up examination and imaging 1 year later with cone beam computed tomography (CBCT) revealed recurrence of the lesion. CBCT showed that the lesion was 25.3x14x12.1 mm in size, the bone mass originated from left lingual mandibular region and the formation of reactive spicula produced by the lesion (Fig. 2). Total excision of the lesion and a margin of surrounding uninvolved bone was performed. It was diagnosed again as intraosseous cavernous hemangioma (Fig. 3).

## Discussion

Benign vascular anomalies can be broadly classified as hemangiomas (vasoproliferative tumors) and vascular malformations. Vascular anomalies often affect the soft tissues of the maxillofacial region but are rarely found in visceral organs or bone. The diagnosis and management of vascular lesions continue to present diagnostic and therapeutic challenges to all. This is in part because of lack of agreement regarding the nosology and classifications of the lesions. Many authors use the term hemangioma to describe or qualify vascular malformations and vascular anomalies, whereas others continue to use the term cavernous hemangioma for venous malformation and port-wine stain for capillary malformation, venous malformation, and arteriovenous malformations (AVM) (10-13).

AVMs are a complex network of intercommunicating arterial and venous structures. The presence of nerve in AVM provides an additional diagnostic criterion that can be readily used to differentiate AVMs from hemangiomas (14).

Hemangiomas are benign vascular neoplasms characterized by an abnormal proliferation of blood vessels. They may occur in any vascularized tissue including skin, subcutaneous tissue, muscle, and bone. The microscope appearance has two major classifications; cavernous and capillary types. The cavernous hemangioma is composed of large thin-walled vessels and sinusoids lined with a single layer of endothelium. However, a small fine vascular network filled with blood forms the capillary hemangioma. Capillary hemangiomas are usually present at birth. In contrast, most cavernous hemangiomas occur in adult hood and almost all intraosseous hemangiomas of the facial skeleton are to be cavernous type (1,6,10,11). In this report the presented mandibular intraosseous hemangioma case was also a 28-year-old adult patient.

The pathogenesis of intraosseous hemangiomas remains unknown. Nonetheless, local trauma may be a cause, because many patients with intraosseous hemangiomas have had a history of local trauma (4), but in our patient, there was no such predisposition for intraosseous hemangioma development.

As intraosseous hemangioma tends to grow very slowly, it remains clinically silent until the tumor becomes large. The radiographic appearance of central hemangioma is not pathognomonic and can simulate many other bone lesions. In this case; the formation of reactive spicula produced by the lesion that was observed in the occlusal radiography is the classic radiographic feature for the tumor (10,11). Preoperative radiological studies include conventional radiography, CT and magnetic resonance imaging (MRI) (10,12). CT is considered the most useful imaging technique because of its unique characterization of trabecular and cortical details, showing the honey comb appearance. MRI is an effective method for identifying the depth of tumor extension into the soft tissues (2,12,13). In our case, CBCT scans revealed a mandibular mass with the formation of reactive spicula, which is characteristic to intraosseous hemangioma.

Hemangiomas should be considered in the differential diagnosis of multilocular lesions involving the body of the ramus and body of the mandible. The differential diagnoses for intraosseous cavernous hemangioma include AVM, osteoma, langerhans cell histiocytosis, fibrous dysplasia, dermoid tumor and multiple myeloma (9-11). Histologically; osteomas may be of two types: compact and cancellous. Periosteal osteomas are mostly cancellous type which is characterized by bony trabeculae and fibro-fatty marrow enclosing osteoblast with an architecture resembling mature bone. In cavernous hemangioma, microscobic examination shows many dilated, thin walled vascular spaces lined by benign endothelial cells containing a large number of red blood cells rather than fibrofatty marrow space. Fibrous connective tissue stroma was seen supporting the spaces interspersed among the trabeculae (6,14). The presence of arteries, arterioles, or both as an integral part of the lesions (as shown by elastic tissue stains) is often used as a diagnostic criterion for differentiating AVMs from hemangiomas.In most cases soft tissue changes such as blood discharge, bluish discoloration, mobility of the teeth suggest a vascular lesion. When a hemangioma produces a sunray spiculated bone pattern at its periphery, the appearance may be difficult to differentiate from an osteogenic sarcoma (10,11,14). In our case, it can be seen many dilated, thin-walled vascular spaces lined by benign endothelial cells separated by bone septum and oedematous-fibrous stroma supporting the bloodfilled vascular spaces.

The most effective treatment to prevent recurrence of intraosseous hemangiomas is to remove the tumor completely without any functional deficit, cosmetic deformity, or significant tissue loss (8). The biopsy and surgery of the lesion should be done cautiously because of the risk of severe bleeding. In the past, radiotherapy and sclerotherapy were the treatment of choice. Radiotherapy may only be reserved for cases in which surgery is not feasible due to the adverse effects such as telangiectasia, retardation of growth of bones and teeth, tissue necrosis, and malignant degeneration. Embolization and sclerotherapy are sometimes performed, but the efficacy is palliative in many cases. Preoperative embolization has been reported to reduce the risk of intraoperative severe bleeding. In contrast, other reports have shown that vascular manipulation is not necessary if en bloc excision can be performed with a sufficient margin (1,8,10-12).In our case, we assessed by preoperative CBCT scans that complete tumor excision with sufficient margins was possible, and could be performed without severe bleeding.

The diagnosis of facial bone hemangiomas is somewhat difficult to make, because the lesion is usually asymptomatic, the most affected patients are women in their fourth and fifth decades (3,5,9), but in this report we presented a male patient on the third decade of life with intraosseous cavernous hemangioma. Early diagnosis of intraosseous hemangioma is essential both for preventing uncontrollable haemorrhage and even death during biopsy or surgery and to a lesser cosmetic deformity. Therefore, dentists should be aware of the radiological features of this vascular bone lesion (10,11). Differential diagnosis of the lesion could be made after the histopathological evaluation in this case, thus when a bone hard, well-shaped mass was seen in the mandible, the possibility of intraosseous hemangioma must be remembered and before surgical procedure detailed radiographic evaluation should be performed.
